# AttentionDDI: Siamese attention-based deep learning method for drug–drug interaction predictions

**DOI:** 10.1186/s12859-021-04325-y

**Published:** 2021-08-21

**Authors:** Kyriakos Schwarz, Ahmed Allam, Nicolas Andres Perez Gonzalez, Michael Krauthammer

**Affiliations:** 1 Department of Quantitative Biomedicine, University of Zurich, Schmelzbergstrasse 26, 8006 Zurich, Switzerland; 2 Biomedical Informatics, University Hospital of Zurich, Zurich, Switzerland

**Keywords:** Drug–drug interactions, Side effects, Prediction, Deep learning, Attention

## Abstract

**Background:**

Drug–drug interactions (DDIs) refer to processes triggered by the administration of two or more drugs leading to side effects beyond those observed when drugs are administered by themselves. Due to the massive number of possible drug pairs, it is nearly impossible to experimentally test all combinations and discover previously unobserved side effects. Therefore, machine learning based methods are being used to address this issue.

**Methods:**

We propose a Siamese *self-attention* multi-modal neural network for DDI prediction that integrates multiple drug similarity measures that have been derived from a comparison of drug characteristics including drug targets, pathways and gene expression profiles.

**Results:**

Our proposed DDI prediction model provides multiple advantages: (1) It is trained end-to-end, overcoming limitations of models composed of multiple separate steps, (2) it offers model explainability via an *Attention* mechanism for identifying salient input features and (3) it achieves similar or better prediction performance (AUPR scores ranging from 0.77 to 0.92) compared to state-of-the-art DDI models when tested on various benchmark datasets. Novel DDI predictions are further validated using independent data resources.

**Conclusions:**

We find that a Siamese multi-modal neural network is able to accurately predict DDIs and that an *Attention* mechanism, typically used in the Natural Language Processing domain, can be beneficially applied to aid in DDI model explainability.

**Supplementary Information:**

The online version contains supplementary material available at 10.1186/s12859-021-04325-y.

## Background

Polypharmacy, the concurrent administration of multiple drugs, has been increasing among patients in recent years [1–3]. When administering multiple drugs, interactions might arise among them, often termed drug–drug interactions (*DDI*). The intended effect of a drug may therefore be altered by the action of another drug. These effects could lead to drug synergy [4], reduced efficacy or even to toxicity [5]. Thus, DDI discovery is an important step towards improved patient treatment and safety.

It is almost impossible to conduct an empirical assessment of all possible drug pair combinations and test their propensity for triggering DDIs. Computational approaches have addressed this issue by enabling the testing of large number of drug pairs more efficiently. For instance, *DeepDDI* [6], a multilabel classification model, takes drug structure data as input along with drug names, in order to make DDI predictions in the form of human-readable sentences. Another model, *GENN* [7], a graph energy neural network, puts a focus on DDI types and estimates correlations between them. *NDD* [8] utilizes multiple drug similarity matrices, which are combined by Similarity Network Fusion (*SNF*) and finally fed through a feed-forward network for classification. Similarly, *ISCMF* [9] performs matrix factorization on the known DDIs in order to calculate latent matrices which are used for predictions. It utilizes the same *SNF*-fused matrix as to constrain this factorization.

The above mentioned solutions come with some drawbacks. First, there is a plethora of drug feature information available for many approved drugs, including chemical structure, side effects, targets, pathways, and more. However, current DDI prediction solutions often only take advantage of a small subset of these features, particularly drug chemical structure features, due to their broad availability. Other current model limitations include low interpretability and/or the fact that they consist of multiple separate steps (i.e., cannot be trained end-to-end). A novel solution should preferably offer a mechanism to tackle those drawbacks simultaneously.

To this end, we introduce *AttentionDDI*, a Siamese *self-attention* multi-modal neural network model for DDI prediction. Our model is inspired by and adapts ideas from Attention-based models (i.e., Transformer network) [10] that showed great success particularly in the Natural Language Processing (*NLP*) domain. Our model 1) is trained end-to-end, 2) offers model explainability and 3) achieves similar or better prediction performance compared to state-of-the-art DDI models when tested on various benchmark datasets.

## Results

*Model evaluation* In order to evaluate the performance of our approach in predicting drug–drug interactions, we focused on four distinct benchmark datasets broadly used in the literature [8, 9, 11–13]. These four datasets consist of one or more drug similarity matrices describing multiple drug characteristics such as chemical structure and side effects. These datasets are explained in detail in the Methods section and Additional file 1, and are henceforth referenced as DS1, DS2 and DS3 (the last one with two variants called CYP and NCYP). The usage of these datasets for comparing our model with previously released models allows for fair benchmarking and reproducibility of our work.

*Evaluation results* We compared our model *AttentionDDI* (the full version and two variants thereof) against state-of-the-art models reported in the literature, as shown in Table 1. Overall, our model achieves similar or better prediction performance when tested on the above mentioned benchmark datasets.

**Table 1:** Model evaluation scores for all datasets. First rank scores and *AttentionDDI* model scores are reported in bold

Model score	DS1		DS2		DS3 (CYP)		DS3 (NCYP)	
	AUC	AUPR	AUC	AUPR	AUC	AUPR	AUC	AUPR
AttentionDDI$\ddag$	**0.954**	**0.924**	**0.986**	**0.904**	**0.989**	**0.775**	**0.986**	**0.890**
AttentionDDI (without siamese)$\ddag$	0.944	0.907	0.965	0.791	0.945	0.277	0.907	0.443
AttentionDDI (without Attention & siamese)$\ddag$	0.944	0.909	0.926	0.596	0.962	0.491	0.953	0.639
NDD*	0.954	0.922	**0.994**	0.890	**0.994**	**0.830**	**0.992**	**0.947**
Classifier ensemble*	0.956	**0.928**	0.936	0.487	0.990	0.541	0.986	0.756
Weighted average ensemble*	0.948	0.919	0.646	0.440	0.695	0.484	0.974	0.599
RF*	0.830	0.693	0.982	0.812	0.737	0.092	0.889	0.167
LR*	0.941	0.905	0.911	0.251	0.977	0.487	0.916	0.472
Adaptive boosting*	0.722	0.587	0.904	0.185	0.830	0.143	0.709	0.150
LDA*	0.935	0.898	0.894	0.215	0.953	0.327	0.889	0.414
QDA*	0.857	0.802	0.926	0.466	0.709	0.317	0.536	0.260
KNN*	0.730	0.134	0.927	0.785	0.590	0.064	0.603	0.235
ISCMF$\dag$	0.899	0.864	–	–	0.898	0.767	0.898	0.792
Classifier ensemble$\dag$	**0.957**	0.807	–	–	0.990	0.541	0.986	0.756
Weighted average ensemble$\dag$	0.951	0.795	–	–	0.695	0.484	0.974	0.599
Matrix perturbation$\dag$	0.948	0.782	–	–	–	–	–	–
Neighbor recommender$\dag$	–	–	–	–	0.953	0.126	0.904	0.295
Label propagation$\dag$	–	–	–	–	0.952	0.126	–	–
Random walk$\dag$	–	–	–	–	–	–	0.895	0.181

$\ddag$Our model, *scores from [8], $\dag$scores from [9]

**Table 2:** Case studies for the top predictions in DS1

Rank	ID A	ID B	Drug A	Drug B	Interaction
1	DB01194	DB00273	Brinzolamide	Topiramate	The risk or severity of adverse effects can be increased when Topiramate is combined with Brinzolamide
2	DB01589	DB00678	Quazepam	Losartan	The metabolism of Quazepam can be decreased when combined with Losartan
3	DB01212	DB00417	Ceftriaxone	PenicillinV	No interactions
4	DB01586	DB00951	Ursodeoxycholicacid	Isoniazid	No interactions
5	DB01337	DB00565	Pancuronium	Cisatracurium	Pancuronium may increase the central nervous system depressant (CNS depressant) activities of Cisatracurium
6	DB00351	DB00484	Megestrolacetate	Brimonidine	No interactions
7	DB00530	DB00445	Erlotinib	Epirubicin	No interactions
8	DB00458	DB00659	Imipramine	Acamprosate	No interactions
9	DB01586	DB00319	Ursodeoxycholicacid	Piperacillin	No interactions
10	DB00443	DB00333	Betamethasone	Methadone	The metabolism of Methadone can be increased when combined with Betamethasone
11	DB00458	DB00321	Imipramine	Amitriptyline	The metabolism of Amitriptyline can be decreased when combined with Imipramine
12	DB00790	DB00584	Perindopril	Enalapril	The risk or severity of angioedema can be increased when Enalapril is combined with Perindopril
13	DB01059	DB00448	Norfloxacin	Lansoprazole	No interactions
14	DB00571	DB01203	Propranolol	Nadolol	Propranolol may increase the arrhythmogenic activities of Nadolol
15	DB00975	DB00627	Dipyridamole	Niacin	No interactions
16	DB00967	DB01173	Desloratadine	Orphenadrine	Desloratadine may increase the central nervous system depressant (CNS depressant) activities of Orphenadrine
17	DB00222	DB00328	Glimepiride	Indomethacin	The protein binding of Glimepiride can be decreased when combined with Indomethacin
18	DB00193	DB01183	Tramadol	Naloxone	The metabolism of Naloxone can be decreased when combined with Tramadol
19	DB00904	DB00918	Ondansetron	Almotriptan	The risk or severity of adverse effects can be increased when Ondansetron is combined with Almotriptan
20	DB00423	DB00794	Methocarbamol	Primidone	The risk or severity of adverse effects can be increased when Methocarbamol is combined with Primidone

Interaction information from the DrugBank database

**Table 3:** Benchmark datasets

Dataset	$\#$ drugs	Similarity matrices
DS1 [11]	548	Chemical, enzyme, indication, offside effects, pathway, side effects, target, transporter
DS2 [12]	707	Chemical
DS3 [13]	807	ATC, chemical, GO, Ligand, PPI distance, side effects, target

For DS1, our model achieves an AUPR score of 0.924, outperforming the baseline *NDD* model (AUPR 0.922). The best performing model for DS1 is the Classifier ensemble model (AUPR 0.928). For DS2 our model outperforms all models with an AUPR score of 0.904, with NDD coming second with an AUPR score of 0.89. For DS3 with the CYP labels, our model achieves the second best AUPR score of 0.775, surpassed by the baseline model (AUPR 0.830). Of note, most other models perform poorly (AUPR $< 0.5$) on this dataset. Finally, for DS3 with NCYP labels our model (AUPR score of 0.890) outperforms all models except for the *NDD* model (AUPR 0.947).

We further compared *AttentionDDI* (our model) to two model variants where we (1) use Attention only (without siamese architecture) and (2) use neither the Attention nor the siamese components (i.e. deep neural network architecture only). Table 1 shows that the full version of *AttentionDDI* outperforms both variants by a large margin, especially for DS2 and DS3, highlighting the importance of the Attention and siamese components of our model. Moreover, the role of siamese component was further corroborated when assigning more weight to the contrastive loss function (see hyperparameter $\gamma$ in Table 6 and Eq. 16 for more details) that involved using the distance computed between every drug pair representation vectors generated from the siamese architecture in the training datasets.

*Attention weights*

Our model offers model explainability through the Attention scores computed at all layers of the model including the *Feature Attention* layer (Fig. 4). These scores are used to determine the contribution (i.e. weights) of the similarity matrices (i.e. modalities) to each of the drug representation vectors ($z_a, z_b$), namely which drug characteristics lead to better encoding (detailed explanation of this approach is found in Methods).

**Fig. 1:**
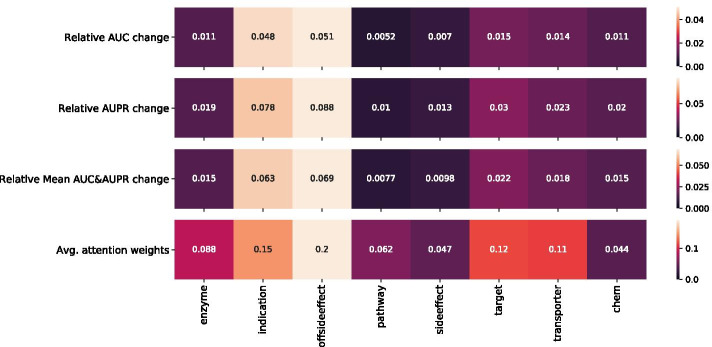
Modality importance using attention scores and masking methods for DS1. First and second rows report modality importance using the masking approach (see Algorithm 1). The values represent the average models’ relative change in AUC and AUPR performance when masking applied to each modality one at a time compared to a base model that has access to all modalities. Third row represents the average between AUC and AUPR average relative change values (i.e. average of values in first and second rows). Fourth row reports average modality importance using Attention score computation (see Eq. 21). The higher the value, the more important the data modality is

**Fig. 2:**
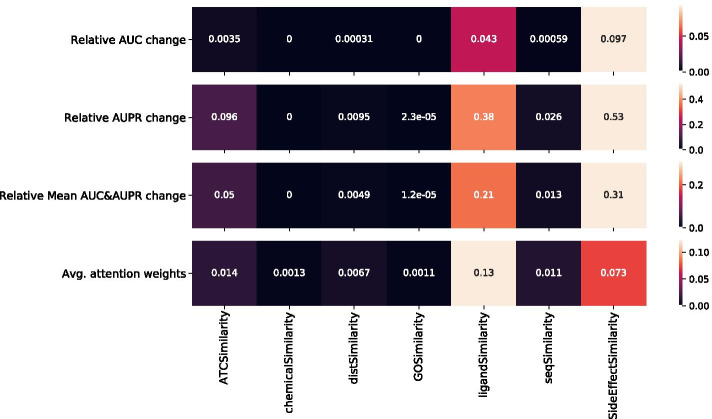
Modality importance using Attention scores and masking methods for DS3 with the CYP labels

In order to assess the capacity of Attention scores for assessing modality importance, we compared the Attention weights to results from an orthogonal method based on modality masking. The latter approach assesses modality importance by masking each modality one at a time and computing the model’s relative change in performance (AUC and AUPR), compared to a base model that has access to all modalities.

Figure 1 depicts the relative change of AUC and AUPR performance compared to the computed Attention weights for DS1. There is an overall agreement between both methods in determining the top-3 modalities (i.e. similarity matrices) contribution where *offsideeffect* and *indication* are weighted more with an average of 0.2, 0.15 scores respectively.

**Fig. 3:**
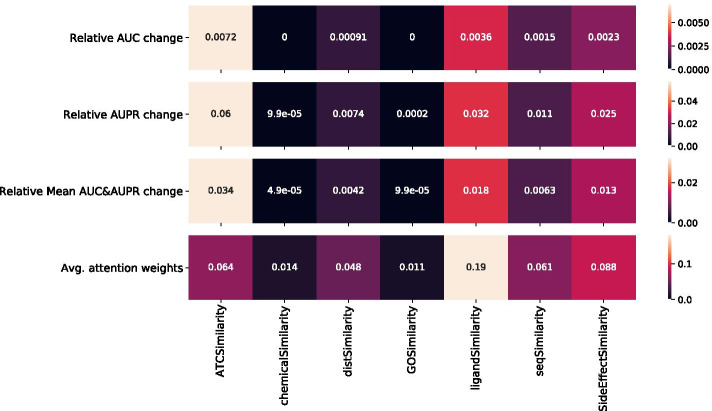
Modality importance using Attention scores and masking methods for DS3 with the NCYP labels

In the DS3 dataset, for both the CYP and NCYP labels, the top-3 ranked similarity matrices were *ligandSimilarity*, *sideeffectSimilarity* and *ATCSimilarity*, as shown in Figs. 2 and 3. Both the relative change in AUC and AUPR, and the Attention score method, overlap in determining the top-3 modalities (i.e. similarity matrices) contribution, thus also illustrating an agreement between both methods.

*Case Studies* To further test the efficiency of our model, we investigated the top predictions of our model through an external drug interaction database, DrugBank [14], which contains DDIs extracted from drug labels and scientific publications. We focused on the DS1 dataset, which links drug similarities to external drug IDs and therefore can be used for external validation. From DS1, we selected the top 20 novel predictions (“false positives” according to the DS1 labels) with the highest interaction probabilities from our model, *AttentionDDI*. In Table 2 we list those drug pairs along with the interaction information from DrugBank. We found that 60% of those top predictions were externally confirmed as known drug pair interactions.

**Table 4:** Labels for each dataset

Dataset	$\#$drugs	$\#$drug–drug pairs	$\#$known DDIs	% known DDIs
DS1	548	149,878	48,584	$\sim$ 32
DS2	707	249,571	17,206	$\sim$ 7
DS3 *CYP*	807	325,221	5039	$\sim$ 1.5
DS3 *NCYP*	807	325,221	20,452	$\sim$ 6

**Table 5:** Confusion matrix

		True interactions		
		Positive	Negative	
Predicted	Positive	*TP*	*FP*	$\mathbf{Precision} = TP / (TP + FP)$
Interactions	Negative	*FN*	*TN*	
		$\mathbf{TPR} , \mathbf{Recall} =TP / (TP + FN)$	$\mathbf{FPR} =FP / (FP + TN)$

**Table. 6:** Training hyperparameters

	DS1	DS2	DS3 CYP	DS3 NCYP
# Attention heads (*H*)	2	2	4	2
# transformer units (*E*)	1	1	1	1
Dropout	0.3	0.3	0.45	0.3
MLP embed factor ($\xi$)	2	2	2	2
Pooling mode	attn	attn	attn	attn
Distance	cosine	cosine	cosine	cosine
Weight decay	$1^{-6}$	$1^{-6}$	$1^{-8}$	$1^{-6}$
Batch size	1000	1000	400	1000
# epochs	100	100	200	100
$\gamma$	0.05	0.05	0.05	0.05
$\mu$	1	1	1	1

## Discussion

### End-to-end solution

In this work, we presented an end-to-end architecture that utilizes an Attention mechanism to train a DDI prediction model. When looking at the DDI models reported in the literature, most of them consist of separate steps for model training. For example, the two competing baseline models (*NDD* and *ISCMF*) consist of multiple cascaded steps such as (1) matrix selection/filtering, (2) matrix fusion, and (3) classification that are optimized separately during model training. Preferably, the matrix selection would be informed by the classification goal. However, the first two steps (matrix filtering and fusion) are independent from classification and therefore not informed by the model training task. In contrast, our model uses a holistic approach in which all computational steps are connected and optimized while minimizing the loss function of our classifier. Consequently, our model is able to optimize the input information for DDI predictions at every computational step.

### Explainability

Along with DDI predictions, our model makes it possible to gain additional information on modality importance. When looking at the relative importance of the Attention weights, the phenotypic information such as drug indication and offside effect similarities were ranked higher than the lower level information (chemical) in DS1 (Fig. 1). This agrees with the conclusion in [15] that phenotypic information is more informative for DDI prediction compared to biological and chemical information. In DS3 for both the CYP as well as the NCYP labels, the phenotypic and biological information contributed more for the model’s prediction, as independently verified by our masking experiments.

### Evaluation of model components

We explored the contribution of the siamese architecture and Attention to model performance. Comparing two model variants, an (1) Attention only model (i.e. without siamese architecture) and a (2) deep neural network model (i.e. without Attention and siamese components), to the full *AttentionDDI* model, we found that the latter vastly outperformed the model variants on DS2 and DS3 (see Table 1). These results provide evidence for the importance of both components (i.e. Attention and siamese architecture) for our model’s state-of-the-art performance.

### Weighing the loss functions

Our model’s loss function was defined by a linear combination of two loss functions: (1) the negative log-likelihood loss (NLL) and (2) the contrastive loss (Eq. 16). The contribution of the NLL loss was included as a standardized loss used in the classification tasks. On the other hand, the contrastive loss focuses on minimizing the intra-class distances (among positive or negative samples) and maximize the inter-class distances (between positive and negative samples).

In our experiments, the importance of the contrastive loss over the NLL loss became evident especially for DS3 datasets. For DS1 and DS2, a uniform weight between both losses would result in a slight decrease of performance as opposed to biasing the weights towards contrastive loss as reported in the manuscript. However, for the DS3 dataset, weighing heavily the contrastive loss was important for achieving the high performance reported in the results section. This could be an indication that the positive and negative samples (that lead to drug interactions or not) are in close distance to each other and not well separated. In such a case, the contrastive loss would assist in better separating those samples and hence improve model performance. This was pronounced in the case of the DS3 dataset, where the proportions of positive samples are low ($\sim$ 1.5% for CYP, $\sim$ 6% for NCYP).

## Conclusions

DDIs have important implications on patient treatment and safety. Due to the large number of possible drug pair combinations, many possible DDIs remain to be discovered. Thus, DDI prediction methods, and particularly computational methods, can aid in the accelerated discovery of additional interactions. These results are valuable for healthcare professionals that aim at finding the most effective treatment combinations while seeking to minimize unintended drug side effects.

In this paper, we present a novel DDI prediction solution which employs *Attention*, a mechanism that has successfully advanced model performance in other domains (such as *NLP*). We demonstrated that *Attention* based models can be successfully adapted to multi-modal biological data in the DDI domain with increased DDI prediction performance over various benchmark datasets and enhanced model explainability.

## Methods

### Benchmark datasets

In order to predict interactions between drugs, we focused on specific benchmark datasets listed in Table 3. Our model, *AttentionDDI*, and two competitive baseline models, *NDD* [8] and *ISCMF* [9], are all built to take advantage of the multi-modality contained in those datasets. Each dataset consists of one or more drug similarity matrices as described in Table 3 and in more detail in the Additional file 1. Those matrices are calculated based on the following drug characteristics: chemical structure, targets, pathways, transporter, enzyme, ligand, indication, side effects, offside effects, GO terms, PPI distance, and ATC codes. The datasets have been previously used by multiple other studies [8, 9, 11–13].

We obtained the precomputed drug similarity matrices from [8] and further describe them in detail in the Additional file 1. As an example, the *side effects* matrix of the DS1 dataset [11] was constructed as follows: A matrix representing a list of *N* known drugs on the *y*-axis and a list of *M* known side effects on the *x*-axis was created. In this matrix, each row is representing a drug along with its side effects in the $N \times M$ matrix. It is filled with the value 1 in each position where it is known that a drug may cause a specific side effect, 0 otherwise. In this fashion, each drug is represented by a binary feature vector (size *M*). Furthermore, this binary feature matrix was transformed into a similarity matrix using all drug pairs. Given two drugs, $d_a$ and $d_b$, and their binary feature vectors ($u_a$ and $u_b$ $\in [0,1]^M$), their similarity was calculated according to the *Jaccard* score:

$$\begin{aligned} J(u_a,u_b) = M_{11} / (M_{01} + M_{10} + M_{11}),\quad 0 \le J(u_a,u_b) \le 1 \end{aligned}$$

where $M_{01}$ represents the count of positions in $u_a$ and $u_b$ where $u_{ai} = 0$ and $u_{bi} = 1$ ($i \in [1, \ldots , M]$). Similarly, $M_{10}$ represents the count of positions (*i*) in $u_a$ and $u_b$ where $u_{ai} = 1$ and $u_{bi} = 0$. Lastly, $M_{11}$ denotes the count of positions (*i*) in $u_a$ and $u_b$ where $u_{ai} = 1$ and $u_{bi} = 1$. This similarity measure is calculated for each drug pair resulting in a $N \times N$ similarity matrix.

Additionally to the above mentioned matrices, we calculated the Gaussian Interaction Profile (GIP) similarity matrix (according to [16]) based on the interaction labels of each dataset (Table 4). Therefore, in addition to the similarity features listed in Table 3, the GIP of each dataset label matrix is also utilized as a further similarity feature. This method assumes that drugs with resembling existing labels (DDIs) are expected to have comparable novel interaction predictions.

DS2 and DS3 were generated by similar approaches. The description of the similarity matrices construction can be found in [11–13] for DS1, DS2 and DS3 datasets respectively and further summarized in the Additional file 1.

### Database DDI labels

In a supervised classification setting, labels of known drug–drug interactions are required in the form of a binary matrix with the same dimensions ($N \times N$) as the input similarity matrices (Table 4). For example, the labels in DS1 were provided by the *TWOSIDES* database [17].

Notably, the DS3 dataset labels are split based on whether the DDIs result from a shared CYP metabolizing enzyme (*CYP*) or not (*NCYP*). This separation was made on the grounds that CYPs are major enzymes involved in $\sim$ 75% of the total drug metabolism. As an example, one drug would inhibit a specific CYP enzyme which also metabolizes another drug, therefore triggering a CYP-related DDI. This separation of CYP labels can affect the model training and predictability, as the positive labels are way outnumbered by the negative ones (Table 4).

The known DDIs in these label matrices have the label value 1. Label 0, however, does not guarantee the absence of drug interactions for the given drug pair. An interaction in this case, may not have been observed yet, or may not have been included in the specific DDI database.

### Model evaluation

The model performance is evaluated based on standardized classification metrics. We included (1) *AUC-ROC* and (2) *AUC-PR*. For consistency with previous studies, we denote them by *AUC*, *AUPR* from now on. These scores are composed according to the definitions in Table 5.

*AUPR* is the Area Under the Precision-Recall curve and is considered the fairer measure [8] especially when class imbalance (i.e., unequal label distribution) is prevalent in the dataset. This is notably the case when the number of positive samples (labels with value 1) and the number of negative samples (0 s) are significantly imbalanced. Given the low proportions of positive samples (Table 4) this is the main performance measure we focus on for the model evaluation. We furthermore computed the *AUC* as standard classification metric. *AUC* is the Area Under the TPR-FPR Curve, where TPR (also Recall) is the True Positive Rate and FPR is the False Positive Rate, as defined in Table 5.

### Baseline model

We compared our model to multiple baseline models found in the literature with special focus on *NDD* [8] that showed high performance on DDI prediction (as reported by the authors). *NDD* consists of three parts: (1) In a first step, the similarity matrices are filtered based on matrix entropy scores. This aims at basing the classification only on the most informative similarity matrices and therefore excluding less informative ones using *handcrafted* heuristics. (2) In a second step, the remaining similarity matrices are merged into one matrix through the *SNF* method (i.e., using similarity network fusion algorithm) [18]. (3) Finally, the fused matrix is used as input to a feed-forward classifier network which outputs binary DDI predictions.

We re-implemented (to the best of our ability) *NDD* using the *PyTorch* deep learning library [19] for the purpose of reproducing the baseline model results. However, we were not able to reproduce the model results reported in [8] especially for DS2 and DS3 datasets. Therefore, we report the performance values cited by the author in their article [8, 9].

### AttentionDDI: model description

**Fig. 4:**
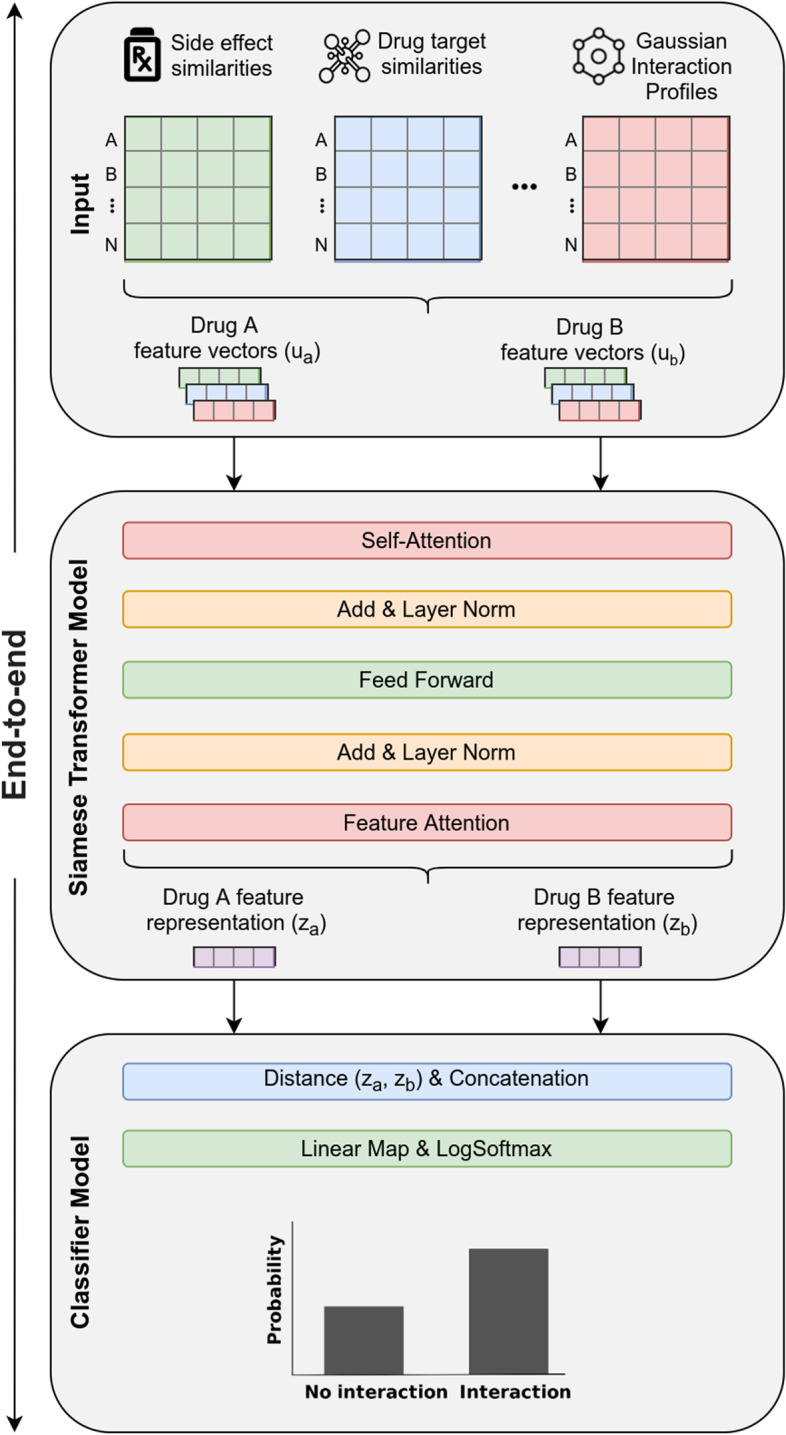
AttentionDDI model architecture. (1) The sets of drug pair feature vectors $(u_a,u_b)$ from each similarity matrix are used as model input, separately for each drug. (2) A *Transformer*-based Siamese encoder model generates new drug feature representation vectors for each drug. First, by applying learned weights (through *Self-Attention*) to the drug feature vectors. Then, by non-linearly transforming the weighted feature vectors by a feed-forward network. Finally, a *Feature Attention* pooling method aggregates the transformed feature vectors into a single feature vector representation for each drug ($z_a$ or $z_b$ respectively). 3) A separate classifier model concatenates the encoded feature vectors $z_a,z_b$ with their distance (*euclidean* or *cosine*). Lastly, through affine mapping of the concatenated drug pair vectors followed by *Softmax* function, a drug-interaction probability distribution is generated for each drug pair

We constructed a Siamese *multi-head* *self-Attention* *multi-modal* neural network model (Fig. 4) adapting the Transformer architecture to model our DDI problem.

*Siamese model* Our model is a Siamese neural network [20] designed to use the same model weights for processing in tandem two different input vectors. In our case the drug similarity features of each drug pair $(d_a, d_b)$ are encoded in parallel in order to learn improved latent vector representations. They are used in a later stage for computing a distance/similarity between both vectors.

*Transformer architecture* Our model architecture adapts the *Transformer* network [10] that uses *multi-head* *self-attention* mechanism to compute new latent vector representations from the set of input vectors while being optimized during training for our *DDI* prediction problem. It consists of:

1. An *Encoder* model, which takes as input a set of drug similarity feature vectors and computes a new (unified) fixed-length feature vector representation.
2. A *Classifier* model, which given the new feature vector representations, generates a probability distribution for each drug pair, indicating if this drug pair is more likely to interact or not.

*Input vectors* Our model is trained on each benchmark dataset (i.e., DS1, DS2 and DS3) separately. There are one or more similarity matrices in a given dataset and *N* distinct number of drugs. Furthermore, there are $$K = \left( {\begin{array}{c}N\\ 2\end{array}}\right)$$ drug pair combinations in every dataset. For a drug pair $(d_a,d_b)$ in a dataset *D*, the drug feature vectors $(u_a,u_b)$ each represent a set of input feature vectors extracted from corresponding similarity matrix $\{S_1, S_2, \ldots , S_T\} \in D$ (including GIP) in dataset *D*. Each set (i.e., $u_a$ and $u_b$) is used as model’s input for each drug separately where *T* feature vectors are processed. For instance, a dataset with three similarity matrices (including GIP) would have two sets of three input vectors (Fig. 4) for each drug pair:

$$\begin{aligned} u_a = \{S_1^{d_a}, S_2^{d_a}, S_3^{d_a}\},\quad u_b = \{S_1^{d_b}, S_2^{d_b}, S_3^{d_b}\} \end{aligned}$$

### Encoder model

For each drug pair $(d_a,d_b)$ the sets of drug feature vectors $(u_a,u_b)$ go through the Encoder separately, in parallel (hence, Siamese model). The Encoder consists of multiple layers. Initially, the input vectors go through a *Self-Attention* layer that aims at generating improved vector encoding (i.e., new learned representation) while optimizing for the target task (i.e., classification in our setting). During this step, the drug feature vectors are weighted according to how strongly they are correlated to the other feature vectors of the same drug. Subsequently, those weighted vectors are fed into a feed-forward network in order to calculate new feature vector representations via non-linear transformation. Lastly, the encoded feature vector representations are passed through a *Feature Attention* layer which aggregates the learned representations, i.e., pools across similarity type vectors. The Encoder then outputs the two separate drug representation vectors $(z_a,z_b)$ which are then fed into the Classifier model. Additionally, there are *Add + Normalize* layers (i.e., residual connections and normalization) after the *Self-Attention* and *Feed-Forward* layers which are used for more efficient training. To summarize, the encoder consists of the following layers in this order: *Self-Attention*, *Add + Normalize*, *Feed-Forward*, *Add + Normalize*, *Feature Attention*.

#### Self-attention layer

We followed a multi-head self-attention approach where multiple single-head self-attention layers are used in parallel (i.e., simultaneously) to process each input vector in set *u* (i.e., $u_a$ for drug $d_a$). The outputs from every single-head layer are concatenated and transformed to generate a fixed-length vector using an affine transformation. The single-head self-attention approach [10] performs linear transformation to every input vector using three separate matrices: (1) a queries matrix $W_{query}$, (2) keys matrix $W_{key}$, and (3) values matrix $W_{value}$. Each input $u_t$ where *t* indexes the feature vectors in *u* (i.e., set of input feature vectors for a given drug extracted from similarity matrices $\{S_1, S_2, \ldots , S_T\} \in D$) is mapped using these matrices to compute three new vectors (Eqs. 1, 2, and 3)

1 $$\begin{aligned} q_t= & {} W_{query} u_t \end{aligned}$$

2 $$\begin{aligned} k_t= & {} W_{key} u_t \end{aligned}$$

3 $$\begin{aligned} v_t= & {} W_{value} u_t \end{aligned}$$

where $W_{query}$, $W_{key}$, $W_{value}$ $\in {\mathbb {R}}^{d'\times d}$, $q_t$, $k_t$, $v_t$ $\in {\mathbb {R}}^{d'}$ are query, key and value vectors, and $d'$ is the dimension of the three computed vectors respectively. In a second step, Attention scores are computed using the pairwise similarity between the query and key vectors for each input vector $u_t$ in the input set *u*. The similarity is defined by computing a scaled dot-product between the pairwise vectors. For each input vector, we compute Attention scores $\alpha _{tl}$ representing the similarity between $q_t$ and vectors $k_l$ $\forall l \in [1, \dots , T]$ where *T* representing the number of vectors in the input set *u* (Eqs. 4, 5) and then normalized using *softmax* function. Then a weighted sum using the Attention scores $\alpha _{tl}$ and value vectors $v_l$ $\forall l \in [1, \dots , T]$ is performed (Eq. 6) to generate a new vector representation $r_t \in {\mathbb {R}}^{d'}$ for the input vector $u_t$. This process is applied to every input vector in the input set *u* to obtain a new set of input vectors ${\underline{R}} = \{r_1,r_2, \ldots , r_{T}\}$.

4 $$\begin{aligned} \alpha _{tl}= & {} \frac{\exp {(score(q_t, k_l))}}{\sum _{l=1}^{T}\exp {(score(q_t,k_l))}} \end{aligned}$$

5 $$\begin{aligned} score(q_t, k_l)= & {} \frac{{q_t}^\top k_l}{\sqrt{d'}} \end{aligned}$$

6 $$\begin{aligned} r_t= & {} \sum _{l=1}^T \alpha _{tl}v_l \end{aligned}$$

In a multi-head setting with *H* number of heads, the queries, keys and values matrices will be indexed by superscript *h* (i.e., $W^h_{query}$, $W^h_{key}$, $W^h_{value}$ $\in {\mathbb {R}}^{d'\times d}$) and applied separately to generate a new vector representation $r^h_t$ for every single-head self-attention layer. The output from each single-head layer is concatenated into one vector $r^{concat}_t = concat(r^1_t, r^2_t, \ldots , r^H_t)$ where $r^{concat}_t \in {\mathbb {R}}^{d'H}$ and then transformed using affine transformation (Eq. 7) such that $W_{unify} \in {\mathbb {R}}^{d'\times d'H}$ and $b_{unify} \in {\mathbb {R}}^{d'}$. This process is applied to each position in the set ${\underline{R}}$ to generate a new set of vectors $\underline{{\tilde{R}}} = \{{\tilde{r}}_1,{\tilde{r}}_2, \ldots , {\tilde{r}}_T\}$.

7 $$\begin{aligned} {\tilde{r}}_t = W_{unify} r^{concat}_t + b_{unify} \end{aligned}$$

#### Layer normalization and residual connections

We used residual/skip connections [21] in order to improve the gradient flow in layers during training. This is done by summing both the newly computed output of the current layer with the output from the previous layer. In our setting, a first residual connection sums the output of the self-attention layer ${\tilde{r}}_t$ and the input vector $u_t$ for every feature vector in the input set *u*. We will refer to the summed output by ${\tilde{r}}_t$ for simplicity.

Layer normalization [22] was used in two occasions; after the self-attention layer and the feed-forward network layer with the goal to ameliorate the “covariate-shift” problem by re-standardizing the computed vector representations (i.e., using the mean and variance across the features/embedding dimension $d'$). Given a computed vector ${\tilde{r}}_t$, *LayerNorm* function will standardize the input vector using the mean $\mu _t$ and variance $\sigma _t^2$ along the features dimension $d'$ and apply a scaling $\gamma$ and shifting step $\beta$ (Eq. 10). $\gamma$ and $\beta$ are learnable parameters and $\epsilon$ is small number added for numerical stability.

8 $$\begin{aligned} \mu _t= & {} \frac{1}{d'}\sum _{j=1}^{d'}{\tilde{r}}_{tj} \end{aligned}$$

9 $$\begin{aligned} \sigma ^2_t= & {} \frac{1}{d'}\sum _{j=1}^{d'}({\tilde{r}}_{tj} - \mu _t)^2 \end{aligned}$$

10 $$\begin{aligned} LayerNorm({\tilde{r}}_t)= & {} \gamma \times \frac{{\tilde{r}}_t - \mu _t}{\sqrt{\sigma ^2_t + \epsilon }} + \beta \end{aligned}$$

#### FeedForward layer

After a layer normalization step, a feed-forward network consisting of two affine transformation matrices and non-linear activation function is used to further compute/embed the learned vector representations from previous layers. The first transformation (Eq. 11) uses $W_{MLP1} \in {\mathbb {R}}^{\xi d' \times d'}$ and $b_{MLP1} \in {\mathbb {R}}^{\xi d'}$ to transform input ${\tilde{r}}_t$ to new vector $\in {\mathbb {R}}^{\xi d'}$ where $\xi \in {\mathbb {N}}$ is multiplicative factor. A non-linear function such as $ReLU(z)=max(0,z)$ is applied followed by another affine transformation using $W_{MLP2} \in {\mathbb {R}}^{d'\times \xi d'}$ and $b_{MLP2} \in R^{d'}$ to obtain vector $g_t \in {\mathbb {R}}^{d'}$. A layer normalization (Eq. 12) is applied to obtain ${\tilde{g}}_t \in {\mathbb {R}}^{d'}$.

11 $$\begin{aligned} g_t= & {} W_{MLP2} ReLU(W_{MLP1} {\tilde{r}}_t + b_{MLP1}) + b_{MLP2} \end{aligned}$$

12 $$\begin{aligned} {\tilde{g}}_t= & {} LayerNorm(g_t) \end{aligned}$$

These transformations are applied to each vector in set $\underline{{\tilde{R}}}$ to obtain new set $\underline{{\tilde{G}}} = \{{\tilde{g}}_1, {\tilde{g}}_2, \ldots , {\tilde{g}}_T\}$. At this point, the *encoder* block operations are done and multiple encoder blocks can be stacked in series for *E* number of times. In our experiments, *E* was a hyperparameter that was empirically determined using a validation set (as the case of the number of Attention heads *H* used in self-attention layer).

#### Feature attention layer

The feature Attention layer is parameterized by a *global* context vector *c* with learnable parameters optimized during the training. For a set of input vectors $\underline{{\tilde{G}}} = \{{\tilde{g}}_1, {\tilde{g}}_2, \ldots , {\tilde{g}}_T\}$ (computed in the layer before), Attention scores $\psi _t \forall t \in [1, \ldots , T]$ are calculated using the pairwise similarity between the context vector $c \in {\mathbb {R}}^{d'}$ and the set $\underline{{\tilde{G}}}$ (Eqs. 13, 14). These scores are normalized and used to compute weighted sum of the $\{{\tilde{g}}_1, {\tilde{g}}_2, \ldots , {\tilde{g}}_T\}$ vectors to generate a new *unified* vector representation $z \in {\mathbb {R}}^{d'}$ that is further passed to the classifier layer (Eq. 15).

13 $$\begin{aligned} \psi _{t}= & {} \frac{\exp {(score(c, {\tilde{g}}_t))}}{\sum _{j=1}^{T}\exp {(score(c,{\tilde{g}}_j))}} \end{aligned}$$

14 $$\begin{aligned} score(c, {\tilde{g}}_t)= & {} \frac{{c}^\top {\tilde{g}}_t}{\sqrt{d'}} \end{aligned}$$

15 $$\begin{aligned} z= & {} \sum _{t=1}^T \psi _{t}{\tilde{g}}_t \end{aligned}$$

*Classifier layer* The classifier layer calculates a distance (*euclidean* or *cosine*) between the computed representation vectors $(z_a,z_b)$ and then concatenates them with that distance. Subsequently, through an affine transformation, the concatenated feature vector is mapped to the size of the output classes (i.e., presence or absence of interaction). Finally, a *softmax* function is applied to output the predicted probability distribution over those two classes (i.e. ${\hat{y}}_{(i)}$ for $i^{th}$ drug pair).

### Objective function

We defined the total loss for an *i*th drug pair by a *linear* combination of the negative log-likelihood loss ($L^C$) and the contrastive loss ($L^{Dist}$). The contribution of each loss function is determined by a hyperparameter $\gamma \in (0,1)$. Additionally, a weight regularization term (i.e., $l_2$-norm regularization) applied to the model parameters represented by $\varvec{\theta }$ is added to the objective function (Eq. 16).

16 $$\begin{aligned} L^{Total} = \gamma L^C + (1-\gamma ) L^{Dist}\ + \frac{\lambda }{2}||\mathbb {\varvec{\theta }}||_{2}^{2} \end{aligned}$$

where

17 $$\begin{aligned} l^{C}_{(i)}= & {} - [y_{(i)} log {\hat{y}}_{(i)} + (1-y_{(i)})log(1- {\hat{y}}_{(i)})], y_i \in \{0,1\} \end{aligned}$$

18 $$\begin{aligned} L^{C}= & {} \frac{1}{K}\sum _{i=1}^{K} l^{C}_{(i)} \end{aligned}$$

and

19 $$\begin{aligned} l^{Dist}_{(i)}= & {} {\left\{ \begin{array}{ll} y_i = 1 &{} \frac{1}{2} {Dist}^2_{(i)}\\ y_i = 0 &{} \frac{1}{2} max((\mu - {Dist}_{(i)})^2, 0) \end{array}\right. } \end{aligned}$$

20 $$\begin{aligned} L^{Dist}= & {} \frac{1}{K}\sum _{i=1}^{K} l^{Dist}_{(i)} \end{aligned}$$

$Dist_{(i)}$ represents the computed distance between the encoded vector representations $z_a$ and $z_b$ of $i^{th}$ drug pair, which can be *euclidean* or *cosine* distance. Additionally, $\mu$ is a contrastive loss *margin* hyperparameter.

The training is done using mini-batches where computing the loss function and updating the parameters/weight occur after processing each mini-batch of the training set.

### Model variants

To further assess the contribution of the different components of our model’s architecture, we trained and tested two model variants. The first uses an Attention only model (i.e. without the siamese architecture) where the feature vectors of each drug pair are used as set of input vectors to the model. The second variant disables both the Attention and siamese components, such that it only uses a deep neural network (i.e. feed-forward neural network) where each drug pair feature vectors are simply concatenated and fed to the model. Each model was trained and tested in similar way to the original model (i.e. *AttentionDDI*) on each dataset separately.

### Training workflow

For training, we utilized a 10-fold stratified cross-validation strategy with 10% dedicated for a validation set and hyperparameter tuning (defined in Table 6). For hyperparameter optimization we selected a set of random hyperparameter combinations for each model and then trained them on a random fold (out of 10). Subsequently, we selected the hyperparameters based on the performance of the models on the validation set of the respective fold. Finally, with the selected hyperparameters (Table 6) we retrained each model on all 10 folds. During training, examples were weighted inversely proportional to class/outcome frequencies in the training data. Model performance was evaluated using area under the receiver operating characteristic curve (AUC), and area under the precision recall curve (AUPR). During training of the models, the epoch in which the model achieved the best AUPR on the validation set was recorded, and model state as it was trained up to that epoch was saved. This best model, as determined by the validation set, was then tested on the test split.

### Data modality importance

To determine the importance of each data modality (i.e. similarity matrix) and its contribution to model’s performance, we used two separate methods. The first is based on the Attention scores computed at every layer when a drug pair is passed to the model. Given our *AttentionDDI* model has varying number of Attention heads *H* and varying number of encoder units *E*, we aggregate every computed Attention score matrix $Attn^{[h,e]}$ from the different heads and units and then average it across all layers, where *h* and *e* index the model’s Attention heads and the encoder units respectively. Lastly, the Attention vector computed in the *Feature Attention* layer *featAttn* is used to reweight the averaged Attention matrices as described in Eq. 21.

21 $$\begin{aligned} ModalityImp_{(i)}^{Attn} = featAttn_{i} \cdot \left(\frac{1}{E}\sum _{e=1}^{E}\frac{1}{H}\sum _{h=1}^{H} Attn_{i}^{[h,e]}\right) \end{aligned}$$

where $featAttn_i$ $\in {\mathbb {R}}^{1 \times T}$ and $Attn_i^{[h,e]}$ $\in {\mathbb {R}}^{T \times T}$ for the $i-$th drug pair with *T* number of input modalities (i.e. similarity matrices). For each dataset in this study, the average modality importance vector (i.e $ModalityImp_{avg}^{Attn}$) is computed using all test data in the 10-folds.

The second method for evaluating the input modality importance is based on an masking experiment, where for each fold in the 10-folds of a given dataset, we mask each modality one at a time and compute the model’s relative change in performance (AUC and AUPR), compared to a base model that had access to all modalities. Algorithm 1 describes the procedure in details. The higher the relative change, the more important the removed/masked modality is.
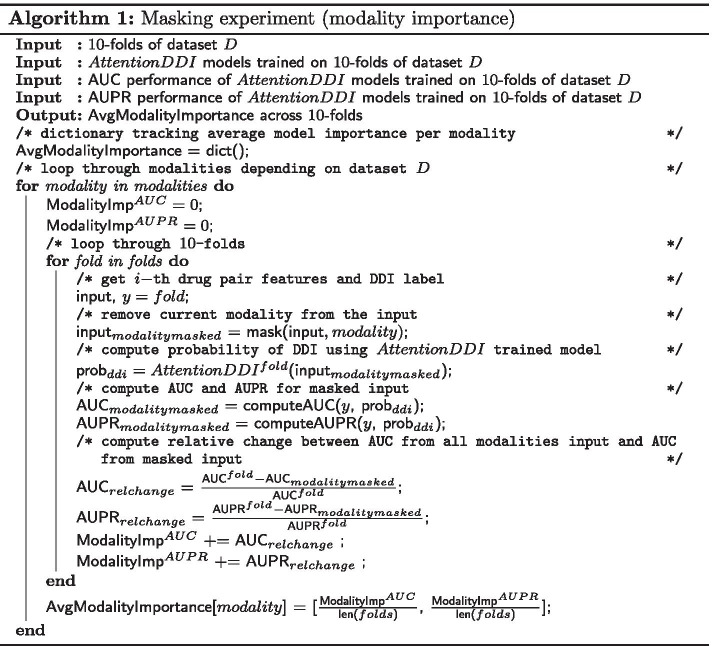


## Supplementary Information

**Additional file 1.** Description of the four datasets used and their corresponding similarity matrices.

## Data Availability

The datasets generated and/or analysed during the current study are available in the Github repository, https://github.com/uzh-dqbm-cmi/side-effects/

## References

[1] Kantor ED, Rehm CD, Haas JS, Chan AT, Giovannucci EL (2015). Trends in prescription drug use among adults in the United States From 1999–2012. JAMA.

[2] Zhang N, Sundquist J, Sundquist K, Ji J (2020). An increasing trend in the prevalence of polypharmacy in Sweden: a nationwide register-based study. Front Pharmacol.

[3] Oktora MP, Denig P, Bos JH, Schuiling-Veninga CC, Hak E (2019). Trends in polypharmacy and dispensed drugs among adults in the Netherlands as compared to the united states. PLoS ONE.

[4] Siniscalchi A, Gallelli L, Avenoso T, Squillace A, De Sarro G (2011). Effects of carbamazepine/oxycodone coadministration in the treatment of trigeminal neuralgia. Ann Pharmacother.

[5] Franceschi A, Tuccori M, Bocci G, Vannozzi F, Di Paolo A, Barbara C, Lastella M, Blandizzi C, Del Tacca M (2004). Drug therapeutic failures in emergency department patients: a university hospital experience. Pharmacol Res.

[6] Ryu JY, Kim HU, Lee SY (2018). Deep learning improves prediction of drug-drug and drug–food interactions. Proc Natl Acad Sci.

[7] Ma T, Shang J, Xiao C, Sun J. GENN: predicting correlated drug–drug interactions with graph energy neural networks. arXiv:1910.02107 [cs, q-bio, stat] (2019). Accessed 15 July 2020

[8] Rohani N, Eslahchi C (2019). Drug–drug interaction predicting by neural network using integrated similarity. Sci Rep.

[9] Rohani N, Eslahchi C, Katanforoush A (2020). ISCMF: integrated similarity-constrained matrix factorization for drug–drug interaction prediction. Netw Model Anal Health Inform Bioinform.

[10] Vaswani A, Shazeer N, Parmar N, Uszkoreit J, Jones L, Gomez AN, Kaiser L, Polosukhin I. Attention is all you need. In: Guyon I, Luxburg UV, Bengio S, Wallach H, Fergus R, Vishwanathan S, Garnett R (eds) Advances in neural information processing systems, vol 30. Curran Associates, Inc.; 2017. pp 5998–6008. http://papers.nips.cc/paper/7181-attention-is-all-you-need.pdf. Accessed 15 July 2020

[11] Zhang W, Chen Y, Liu F, Luo F, Tian G, Li X (2017). Predicting potential drug–drug interactions by integrating chemical, biological, phenotypic and network data. BMC Bioinform.

[12] Wan F, Hong L, Xiao A, Jiang T, Zeng J (2019). NeoDTI: neural integration of neighbor information from a heterogeneous network for discovering new drug–target interactions. Bioinformatics.

[13] Gottlieb A, Stein GY, Oron Y, Ruppin E, Sharan R (2012). INDI: a computational framework for inferring drug interactions and their associated recommendations. Mol Syst Biol.

[14] Wishart DS, Feunang YD, Guo AC, Lo EJ, Marcu A, Grant JR, Sajed T, Johnson D, Li C, Sayeeda Z, Assempour N, Iynkkaran I, Liu Y, Maciejewski A, Gale N, Wilson A, Chin L, Cummings R, Le D, Pon A, Knox C, Wilson M (2018). DrugBank 5.0: a major update to the DrugBank database for 2018. Nucleic Acids Res.

[15] Zhang P, Wang F, Hu J, Sorrentino R (2015). Label propagation prediction of drug–drug interactions based on clinical side effects. Sci Rep.

[16] van Laarhoven T, Nabuurs SB, Marchiori E (2011). Gaussian interaction profile kernels for predicting drug–target interaction. Bioinformatics.

[17] Tatonetti NP, Ye PP, Daneshjou R, Altman RB (2012). Data-driven prediction of drug effects and interactions. Sci Transl Med.

[18] Wang B, Mezlini AM, Demir F, Fiume M, Tu Z, Brudno M, Haibe-Kains B, Goldenberg A (2014). Similarity network fusion for aggregating data types on a genomic scale. Nat Methods.

[19] Paszke A, Gross S, Chintala S, Chanan G, Yang E, DeVito Z, Lin Z, Desmaison A, Antiga L, Lerer A. Automatic differentiation in PyTorch; 2017. Accessed 29 July 2020.

[20] Chicco D. In: Cartwright H (ed) Siamese neural networks: an overview. New York: Springer; 2021. pp 73–94. 10.1007/978-1-0716-0826-5_3 32804361

[21] He K, Zhang X, Ren S, Sun J. In: Deep residual learning for image recognition, vol 2016. IEEE Computer Society; 2016. p. 770–8. 10.1109/CVPR.2016.90. arXiv:1512.03385.

[22] Ba JL, Kiros JR, Hinton GE. Layer normalization; 2016. arXiv:1607.06450.

