# Supplementary material for: AttentionDDI: Siamese attention-based deep learning method for drug–drug interaction predictions
Source: BMC Bioinformatics. 2021 Aug 21;22:412. doi: 10.1186/s12859-021-04325-y (PMC8379737; doi:10.1186/s12859-021-04325-y)
Supplement: Supplementary file 1 — Additional file 1. Description of the four datasets used and their corresponding similarity matrices. [file 12859_2021_4325_MOESM1_ESM.pdf]

RESEARCH

# AttentionDDI: Siamese Attention-based Deep Learning method for drug-drug interaction predictions - Supplementary information

Kyriakos Schwarz[1,2]
, Ahmed Allam[1,2]
, Nicolas Andres Perez Gonzalez[1,2]
and Michael Krauthammer[1,2*]

*Correspondence:
michael.krauthammer@uzh.ch
[1]Department of Quantitative Biomedicine, Schmelzbergstrasse 26, 8006 Zürich, CH
Full list of author information is available at the end of the article

## Sources for similarity measures

In order to compare the performance of our approach with previous models, we used four datasets refered to as DS1, DS2 and DS3 (the last one with two variants, CYP and NCYP).

### DS1 dataset

DS1 contains 548 drugs and eight types of similarity matrices: chemical, target, transporter, enzyme, pathway, indication, side effects and offside effects. The sources for this dataset are referenced in the work of [1] and colleagues.

### DS2 dataset

DS2 contains 707 drugs and one type of similarity matrix: chemical. The sources for this dataset are referenced in [2].

### DS3 dataset

DS3 contains 807 drugs and seven types of similarity matrices: GO, target, ligand, chemical, PPI distance, side effect and ATC. The sources for this dataset are referenced in Gottlieb et al [3].

#### Sources for each similarity matrix

1 **Chemical**: Authors mention they assembled a drug-drug similarity measure for chemical similarity based on Keiser et al 2009 [4]. The authors mention that “Canonical SMILES” of the drug molecules were obtained from DrugBank to later compute hashed fingerprints by using the Chemical Development Kit (CDK) with default parameters. The authors computed the similarity score between two drugs based in the fingerprint using the two-dimensional Tanimoto Score (equivalent to the Jaccard Score).

2 **Ligand**: In order to get this measure, the authors first focused on the drugs' ”Canonical SMILES”. ”SMILES” refers to the Simplified Molecular Input Line System and it is a line notation system used for describing the structure of a chemical substance using ASCII strings. The authors used a given drug's

canonical SMILES within the Similarity Ensemble Approach (SEA) tool, to compare it against a compendium of ligand sets and compute E-values for those ligand sets. As indicated per Gottlieb and colleagues, the SEA tool relates protein receptors based on the chemical 2D similarity of ligand sets affecting their function. To obtain robust drug-drug similarity, the authors queried drugs in the SEA tool using two ligand databases (MLD Drug data report and WOMBAT) and used two methods to compute the drug fingerprint, resulting in four lists that they ultimately unified in a single list. Finally, the similarity was computed as the Jaccard score between the corresponding sets of receptor families.

3 **Side effects**: Drug side effects where obtained by the authors from the online database SIDER. The authors improved that list by side effect predictions based on the drugs' chemical properties. They estimated the similarity between drugs using the Jaccard score between either their known side effects or top 13 predicted side effects in case they are unknown.

4 **Target**: Drug targets were obtained from DrugBank [5], the DCDB [6], Matador [7] and KEGG DRUG [8] databases.

5 **Transporter**: Drug transporters were obtained from DrugBank. The Jaccard score between either the known transporters of drug pairs is defined as their similarity.

6 **Pathway**: KEGG database [8] is an information resource for protein pathways. Drug targets were mapped to KEGG to obtain drug pathways. The Jaccard score was defined as the similarity between two drugs.

7 **Indication**: Indications where obtained from SIDER, an online database with drug indications. As with the previous measures, the authors estimated the similarity between drugs using the Jaccard score between their known indications.

8 **Offside effect**: Off side effects were obtained from the OFF-SIDES database. The OFF-SIDES database (Tatonetti et al. 2012) contains 1332 drugs and 10′093 ”off-label” side effects. The authors estimated similarity using the Jaccard score between their known off side effects.

9 **Enzyme**: Data was collected from DrugBank leading to a list of enzymes that metabolize a particular given drug. Once the list of enzyme per drug was collected, the similarity between drugs was calculated by the Jaccard score between these binary vectors of length 129 (given 129 identified enzymes).

10 **Gene Ontology (GO)**: According to the authors [3], semantic similarity scores between drug targets were estimated using the *csbl.go* R package (Ovaska et al, 2008) selecting the option to use all three ontologies.

11 **PPI distance**: According to the authors [3], in order to estimate the distance between drug-drug targets, the all-pairs shortest path algorithm was used on the human PPI network. These distances were transformed to similarity values by using the formula used in Perlman et al 2011.

12 **ATC**: Authors use the ATC classification system, which categorizes drugs based on the organ they act on, their therapeutic effect and their chemistry. These codes were obtained from DrugBank. As mentioned by the authors [3], in order to define a similarity between ATC terms, they used the semantic similarity algorithm of (Resnik, 1999). Resnik's algorithm associates probabilities

$p(x)$ with all the nodes $x$ (i.e., ATC levels) in the ATC hierarchy by computing the number of levels below $x$. After this step, the algorithm estimates the drug-drug similarity as the maximum over all their common ancestors ATC level $c$ of $-log(p(c))$.

13 **GIP**: See full description in the main manuscript.

**Author details**
[1]Department of Quantitative Biomedicine, Schmelzbergstrasse 26, 8006 Zürich, CH. [2]Biomedical Informatics, University Hospital of Zurich, Zurich, Switzerland.

**References**

1. Zhang, P., Wang, F., Hu, J., Sorrentino, R.: Label Propagation Prediction of Drug-Drug Interactions Based on Clinical Side Effects. Scientific Reports **5**(1), 1–10 (2015). doi:10.1038/srep12339. Accessed 2020-10-13
2. Wan, F., Hong, L., Xiao, A., Jiang, T., Zeng, J.: NeoDTI: neural integration of neighbor information from a heterogeneous network for discovering new drug–target interactions. Bioinformatics **35**(1), 104–111 (2019). doi:10.1093/bioinformatics/bty543. Publisher: Oxford Academic. Accessed 2020-07-15
3. Gottlieb, A., Stein, G.Y., Oron, Y., Ruppin, E., Sharan, R.: INDI: a computational framework for inferring drug interactions and their associated recommendations. Molecular Systems Biology **8**(1), 592 (2012). doi:10.1038/msb.2012.26. Publisher: John Wiley & Sons, Ltd. Accessed 2020-07-15
4. Keiser, M.J., Setola, V., Irwin, J.J., Laggner, C., Abbas, A.I., Hufeisen, S.J., Jensen, N.H., Kuijer, M.B., Matos, R.C., Tran, T.B., *et al.*: Predicting new molecular targets for known drugs. Nature **462**(7270), 175–181 (2009)
5. Wishart, D.S., Knox, C., Guo, A.C., Cheng, D., Shrivastava, S., Tzur, D., Gautam, B., Hassanali, M.: Drugbank: a knowledgebase for drugs, drug actions and drug targets. Nucleic acids research **36**(suppl_1), 901–906 (2008)
6. Liu, Y., Hu, B., Fu, C., Chen, X.: Dcdb: drug combination database. Bioinformatics **26**(4), 587–588 (2010)
7. Günther, S., Kuhn, M., Dunkel, M., Campillos, M., Senger, C., Petsalaki, E., Ahmed, J., Urdiales, E.G., Gewiess, A., Jensen, L.J., *et al.*: Supertarget and matador: resources for exploring drug-target relationships. Nucleic acids research **36**(suppl_1), 919–922 (2007)
8. Kanehisa, M., Goto, S., Furumichi, M., Tanabe, M., Hirakawa, M.: Kegg for representation and analysis of molecular networks involving diseases and drugs. Nucleic acids research **38**(suppl_1), 355–360 (2010)
